# Enhancing the Antibiofilm Activity of β-1,3-Glucanase-Functionalized Nanoparticles Loaded With Amphotericin B Against *Candida albicans* Biofilm

**DOI:** 10.3389/fmicb.2022.815091

**Published:** 2022-05-24

**Authors:** Yulong Tan, Su Ma, Ting Ding, Roland Ludwig, Jintae Lee, Jiaman Xu

**Affiliations:** ^1^Special Food Research Institute, Qingdao Agricultural University, Qingdao, China; ^2^Qingdao Special Food Research Institute, Qingdao, China; ^3^Food Biotechnology Laboratory, Department of Food Sciences and Technology, University of Natural Resources and Life Sciences, Vienna, Austria; ^4^School of Chemical Engineering, Yeungnam University, Gyeongsan, South Korea

**Keywords:** antibiofilm, nanoparticle, chitosan, *Candida*, amphotericin B

## Abstract

*Candida* biofilm-related infections cause increased morbidity and mortality in patients with a reduced immune response. Traditional antifungal therapies have proven to be insufficient as the biofilm matrix acts as a perfusion barrier. Thus, novel methods are required to improve drug delivery and kill *Candida* within the biofilm. In this study, chitosan nanoparticles (CSNPs) loaded with Amphotericin B (AMB), which were functionalized with β-1,3-glucanase (Gls), were fabricated (CSNPs-AMB-Gls), and their antibiofilm activity against *Candida albicans* biofilm was evaluated *in vitro*. Scanning electron microscopy (SEM) and confocal laser scanning microscopy (CLSM) were employed to examine biofilm architecture and cell viability. CSNPs-AMB-Gls inhibited planktonic cell growth and biofilm formation effectively and exhibited the highest efficacy on the removal of a mature biofilm than free AMB or CSNPs-AMB. The created nanoparticles (NPs) were found to penetrate the biofilm so as to directly interfere with the cells inside and disassemble the biofilm matrix. CSNPs-AMB-Gls could also eradicate biofilms from clinical isolates. These results suggest the potential applicability of CSNPs-AMB-Gls for the treatment of *Candida* biofilm-related infections.

## Introduction

*Candida* species are typical pathogenic microorganisms, which have been identified as the fourth most common cause of bloodstream infections in the United States and are associated with high morbidity and mortality (Gottardo et al., 2019; Nani et al., 2019). In addition to public health consequences, *Candida* is also related to food contamination, which reduces the nutritional value of products and leads to foodborne intoxications (Piccinelli et al., 2016; Rajkowska and Kunicka-Styczyńska, 2018). The predominant nosocomial fungal pathogen is *Candida albicans*, which is one of the leading causes of infections known to have a great biofilm-forming ability (Rajendran et al., 2016; Wall et al., 2019). *Candida* cells within biofilms, communities of cells embedded in a matrix, exhibit lower susceptibility to antimicrobials due to an extracellular matrix (Mitchell et al., 2016; Lohse et al., 2017). Even high therapeutic concentrations of existing antifungal drugs prove to be less efficient in inhibiting growth or removing biofilms while the risk of causing serious side effects, such as kidney or liver damage, is increased (Lohse et al., 2017).

Amphotericin B (AMB), a broad-spectrum antifungal drug, is recommended as first-line therapy for fungal-related infections (Benincasa et al., 2011; Vikelouda et al., 2017). However, *C. albicans* in the biofilm shows high resistance to AMB, which can be 1,000-fold less susceptible than planktonic ones. Several lipid-based drug delivery systems have been employed to enhance the therapeutic effect and decrease the toxicity of AMB (Weiler et al., 2008; Nieto et al., 2018). Unfortunately, the widespread use of these new formulations is impeded due to the high production cost (Weiler et al., 2009; Steimbach et al., 2017). Therefore, there is an urgent need to precise the delivery to enhance the efficiency and improve the inhibitory effect of AMB on ergosterol in fungal membranes.

Nanobiotechnology has gained attention in the pharmaceutical and medical fields, and the nano-sizing of antimicrobial agents seems to be a promising treatment for biofilm-related infections. Because nanoparticles (NPs) can more easily penetrate the biofilm matrix due to their size and surface charge (Baelo et al., 2015). It has been reported that both positively and negatively charged NPs could bind to biofilms. Negatively charged NPs could bind to microbial cells with hydrophobic interactions, while positively charged NPs could diffuse into the biofilm through binding to negatively charged biofilm polymers, such as eDNA (Nafee et al., 2014). Therefore, they can interfere with cells inside the biofilm directly (Jamil et al., 2017; Benoit et al., 2019). Moreover, the NP structure can enclose and protect antimicrobials from endogenous and exogenous factors of microbial cells, leading to enhanced bioactivity at the proximity of target cells (Jamil et al., 2016). Chitosan (CS) is a cost-effective biopolymer suitable for the preparation of biocompatible, biodegradable, and non-cytotoxic NPs with inherent antimicrobial and antibiofilm activity (Martinez et al., 2010; Klinger-Strobel et al., 2016). In our previous work, CS nanoparticle (CSNP) has been proven to be a kind of ideal carrier for biofilm treatment (Tan et al., 2018). Furthermore, CSNP is a promising carrier to overcome the poor aqueous solubility of hydrophobic drugs (Trapani et al., 2009; Quiñones et al., 2018).

Increased drug resistance of *C. albicans* in biofilms compared to its planktonic form is mainly due to the self-produced extrapolymeric substance (EPS) matrix (Limoli et al., 2015; Flemming et al., 2016). Moreover, the inactive structure of the biofilm still promotes the adhesion and regeneration of other microorganisms (MacDonald et al., 2000). Therefore, the biofilm matrix itself should also be a target in addition to fungal cells. Recently, EPS-degrading enzymes have been proposed as a new strategy to remove the biofilm and to enhance drug efficacy (Okshevsky and Meyer, 2015; Kimura and Urata, 2016; Klinger-Strobel et al., 2016). As one of the major polysaccharides in the *C. albicans* biofilm matrix, β-1,3-glucan plays an important role in the biofilm structure and cell protection. The enzyme β-1,3-glucanase (Gls) can disrupt the *Candida* biofilm matrix by degrading β-1,3-glucan (Nett et al., 2007). Our previous work also showed that Gls can enhance the efficacy of antimicrobial drugs (De Brucker et al., 2015; Tan et al., 2018). However, to our knowledge, there are no reports showing nanosystems that combine the enzyme specifically degrading the *Candida* biofilm matrix and the antifungal that kills *Candida* within the biofilm.

Therefore, the combination of CSNPs with antimicrobials and enzymes as novel nanoantimicrobials seems to be a plausible approach for the treatment of *Candida* biofilms. In this study, CSNPs were functionalized with Gls and loaded with AMB (CSNPs-AMB-Gls) to combine enhanced antibiofilm activity with biofilm matrix disruption ability.

## Materials and Methods

### Strains, Media, and Reagents

*Candida albicans* DAY 185 was used in the experiments. Three clinical isolates of *C. albicans*, named *C. albicans* BF1, BF2, and BF3, were obtained from the Medical University of Vienna. Each experiment was repeated three times. All of them are able to form strong biofilms, which have been confirmed by the crystal violet method. The strains were cultured in a Yeast Peptone Dextrose (YPD) medium (Sigma-Aldrich, Austria) at 30°C. RPMI 1640 medium (Thermo Fisher Scientific, Waltham, MA, United States) was used for biofilm growth. Gls (lyticase from *Arthrobacter luteus*, ≥2,000 units/mg protein), CS (low molecular weight, degree of deacetylation 75–85%), AMB, and other reagents were purchased from Sigma-Aldrich.

### Formulation of Nanoparticles

Chitosan nanoparticles were prepared as described by the ion gelation method with polyanionic sodium triphosphate (TPP) in our previous work (Pu et al., 2014). CSNP-Gls was prepared with mixing CSNPs [1 mg/ml polybutylene succinate (PBS)] and Gls (100 μg/ml PBS) while stirring overnight at 4°C. NP suspensions were centrifuged (14,000 rpm, 30 min) and freeze-dried eventually. For the preparation of CSNPs-AMB-Gls, CSNPs-Gls suspension (1 mg/ml) was mixed with AMB (100 μg/ml DMSO) and stirred for 24 h. The mixture was centrifuged and lyophilized.

### Characterization of Nanoparticles

The size of NPs was analyzed by dynamic light scattering (DLS). Laser Doppler velocimetry assays were used to determine the zeta potential (ZetaSizer Nano ZS, Malvern Instruments, United Kingdom). Morphological characterization was confirmed by scanning electron microscopy (SEM, JSM 6310, JEOL Ltd., Akishima, Tokyo, Japan).

The CSNPs-Gls suspension (prepared as reported above) was evaluated using a suspension of yeast as the substrate according to the Sigma-Aldrich protocol for Gls activity assay. The loading capacity (LC) of AMB loaded on the NP was estimated by measuring the absorption at 405 nm spectrophotometrically (Spectroscopy, Persee, TU-1810, China) and calculated as follows:


LC=(tAMB-fAMB)/NPs

Where tAMB, fAMB, and NPs, represent total AMB, free AMB, and NP amount, respectively.

### *In vitro* Release

Release kinetics *in vitro* was carried out as follows: 4 ml of CSNPs-AMB-Gls solution (1 mg/ml in a dialysis bag) was placed in 40 ml of PBS with stirring (100 rpm). At any given time point, 4 ml of PBS was taken and replaced with 4 ml of fresh PBS to maintain the sink volume. The amount of AMB in the solution was measured at 405 nm by UV spectrometry (Persee, TU-1810, China).

### Minimum Inhibitory Concentration Assay

About 100 μl of *Candida* (1 × 10^6^ CFU/ml), including *C. albicans* DAY 185 and three clinical isolates of *C. albicans*, was added to wells of 96-well microplates (Thermo Fisher Scientific, Waltham, MA, United States) with different concentrations of free AMB, CSNPs, and CSNPs-AMB-Gls (4, 2, 1, 0.5, 0.25, 0.125, 0.0625, or 0 μg/ml). The microplate was incubated at 37°C for 24 h at 150 rpm. Minimum inhibitory concentration (MIC) was defined as the lowest concentration of AMB at which no visible growth was detected.

### Growth of Biofilms

Biofilms in a 96-well microplate and on silicone platelets were conducted and assayed as previously described (Tan et al., 2018). In brief, *C. albicans* was diluted to 1 × 10^6^ CFU/ml with RPMI 1640 and 100 μl of fungal culture was pipetted into each well of a 96-well microplate. Biofilms were formed at 37°C for 24 h without shaking.

### Penetration of Chitosan Nanoparticles Into Biofilms

Chitosan nanoparticles were labeled with rhodamine B isothiocyanate (CSNPs-RBITC) as previously described (Pu et al., 2014). An autoclaved medical grade silicone platelet (3-mm-diameter, Websinger, Austria) was placed in each well of the 96-well microplate. *C. albicans* DAY 185 biofilms were formed on medical grade silicone platelets for 24 h as described above in the 96-well microplate and mixed with RBITC-CSNPs (100 μg/ml) for 2 h and washed with PBS. The penetration of CSNPs was determined by confocal laser scanning microscopy (CLSM).

### Efficacy on the Biofilm Formation

Biofilms were formed in 96-well microplates as previously described. *C. albicans* DAY 185 cells were incubated with free AMB, a combination of AMB and Gls (AMB+Gls, 2 μg/ml), CSNPs-AMB, and CSNPs-AMB-Gls with different drug concentrations (0, 0.25, 0.5, and 1 μg/ml) for 24 h. Biofilms were washed with PBS and quantified with a cell counting kit-8 (CCK-8, Dojindo Molecular Technologies, Gaithersburg, MD, United States) reduction assay. The absorbance was examined at 450 nm.

### Efficacy on the Mature Biofilm

*Candida albicans* DAY 185 biofilms were formed as described above for 24 h and added with fresh medium containing different concentrations (0, 1, 2, and 4 μg/ml) of free AMB, AMB+Gls, CSNPs-AMB, and CSNPs-AMB-Gls. After another 24 h, the biofilms were washed with PBS and quantified with the CCK-8 reduction assay.

### Efficacy on Biofilms on Medical Silicone Surfaces

Biofilms on silicone platelets were treated with free AMB, AMB+Gls, CSNPs-AMB, and CSNPs-AMB-Gls (4 μg/ml) as described above.

Biofilm architecture was investigated by SEM. Biofilms on silicone platelets were fixed in 3% glutaraldehyde (v/v) in PBS solution overnight at 4°C, and then subjected to serial dehydration with 25, 50, 75, and 100% ethanol for 10 min each. The biofilms were coated with gold and examined by SEM (JSM 6310, JEOL Ltd., Akishima, Tokyo, Japan).

The cells of biofilms were stained with a LIVE/DEAD^®^ BacLight™ Bacterial Viability and Counting kit (L34856, Invitrogen, United States) following the manufacturer’s instructions. Cell viability was observed with CLSM.

### Antibiofilm Activity Against Clinical Isolates

The antibiofilm efficacy of free AMB, AMB+Gls, CSNPs-AMB, and CSNPs-AMB-Gls (2 μg/ml) was tested on three clinical isolates. Biofilms of clinical strains formed in the 96-well microplate were evaluated as described above.

### Statistical Analysis

Statistical analyses of data were determined with GraphPad Prism software program (GraphPad Software, San Diego, CA, United States). Values are given as mean ± standard deviation (SD) of the number of experiments (*n* = 3). Statistical significance was determined by the *t*-test analysis with *p* < 0.05.

## Results

### Characterization of CSNPs-AMB-Gls

**FIGURE 1:**
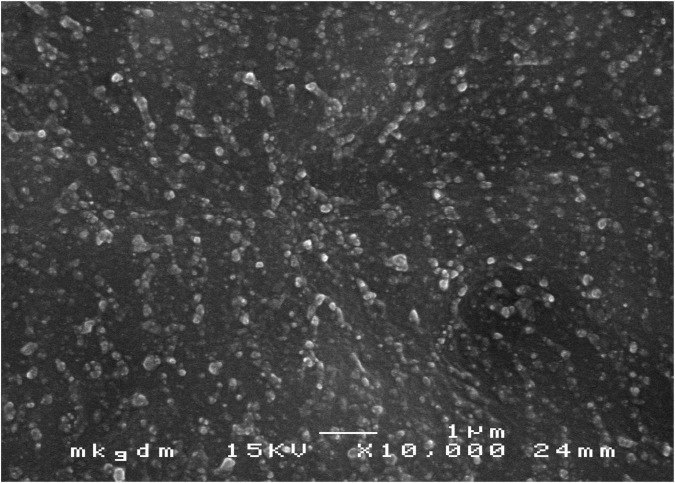
Scanning electron microscopy (SEM) image of chitosan nanoparticles (CSNPs) loaded with amphotericin B (AMB), which were functionalized with β-1,3-glucanase (Gls) (CSNPs-AMB-Gls). Magnification, ×10,000.

Scanning electron microscopy images showed that CSNPs-AMB-Gls was round particles (Figure 1). The average size of CSNPs-AMB-Gls was 174.47 ± 5.12 nm (size PDI 0.17), and the surface zeta potential was +15.84 ± 1.41 mV. The loading capacity of AMB on CSNPs-AMB-Gls was 3.05% ± 0.13%. Gls loaded on the NP-retained Gls activity, the activity of which was 128.6 ± 4.54 U/mg NP.

### *In vitro* Release Studies

**FIGURE 2:**
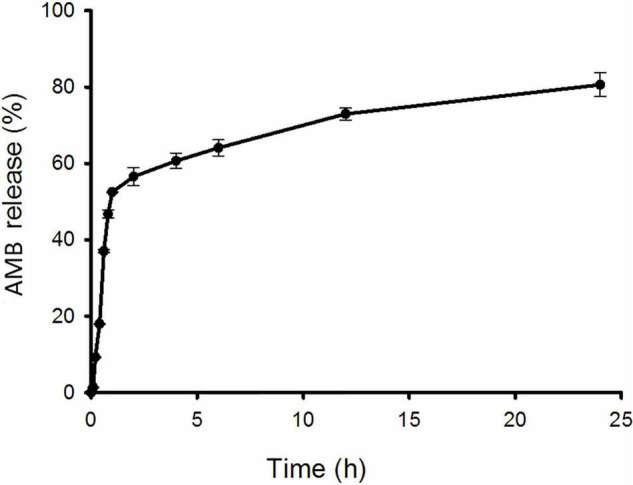
*In vitro* release kinetics of AMB from the nanoparticle (NP). CSNPs-AMB-Gls was placed in polybutylene succinate (PBS) with stirring. The amount of AMB in the solution was measured at 405 nm by UV spectrometry at any given time point.

Figure 2 shows the release profiles of AMB from NPs. CSNPs-AMB-Gls presented an initial burst release phase in the first 1 h: 52.5% of the total AMB was released. Subsequently, the drug release was slowed down. Over 24 h, CSNPs-AMB-Gls showed a sustained release of 80.6% of the total AMB.

### Minimum Inhibitory Concentration Assay

Both free AMB and CSNPs-AMB-Gls were effective against *C. albicans* DAY 185 and the growth of clinical isolates, which showed the same MIC. MIC was 1 μg/ml against *C. albicans* DAY 185 and higher against clinical isolates (2, 2, and 4 μg/ml, respectively). CSNPs alone had no effect on planktonic *Candida* at the concentration used in this work (data not shown). This result suggested that AMB loading in CSNPs did not change the antifungal activity of free AMB to planktonic *Candida*.

### Penetration of Chitosan Nanoparticle Into Biofilms

**FIGURE 3:**
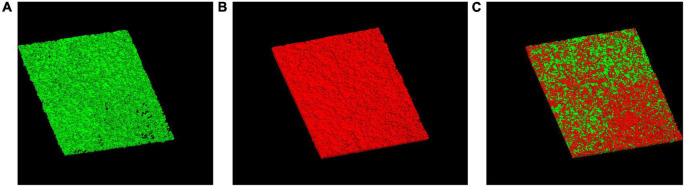
Penetration of CSNPs into biofilms. CSNPs were labeled with rhodamine B isothiocyanate (RBITC) and visualized by confocal laser scanning microscopy (CLSM). SYTO 9-labeled biofilms incubated with CSNPs **(A)**. RBITC-NP penetration into biofilms **(B)**. Overlap **(C)**.

After 2 h treatment with CSNPs, red color (RBITC-NPs) can be observed on the surface and inside the biofilm, suggesting that the penetration of CSNPs into the biofilm (Figure 3).

### Inhibition Activity on Biofilm Formation

**FIGURE 4:**
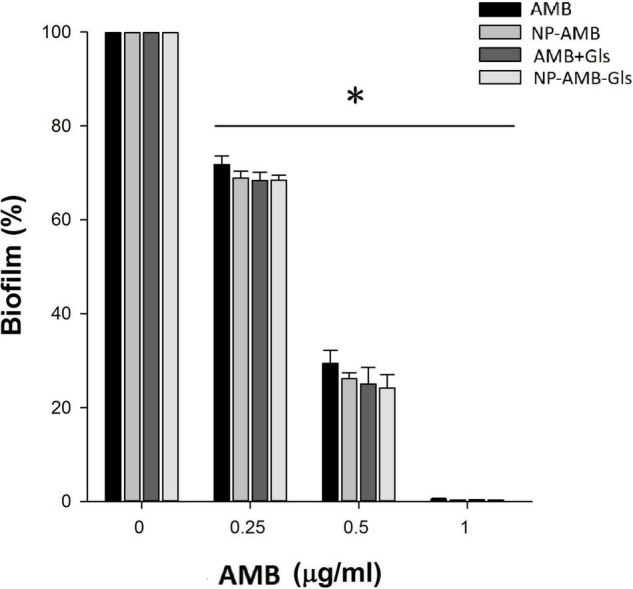
Inhibition efficacy on the biofilm in a 96-well microplate. *Candida albicans* DAY 185 cells were co-incubated with free AMB, AMB+Gls, CSNPs-AMB, and CSNPs-AMB-Gls with different drug concentrations (0, 0.25, 0.5, and 1 μg/ml) for 24 h. The results represent the means and standard deviations (SDs) (error bars), *n* = 3. Statistical significance was determined by the *t*-test analysis. *p < 0.05 for comparison between the untreated and treated groups.

As shown in Figure 4, free AMB, AMB+Gls, CSNPs-AMB, and CSNPs-AMB-Gls showed concentration-dependent biofilm inhibition activity with all tested concentrations. At the concentration of 1 μg/ml, almost no biofilm formations were observed in the treatment with free AMB, AMB+Gls, CSNPs-AMB, and CSNPs-AMB-Gls. At other concentrations, CSNPs-AMB and CSNPs-AMB-Gls showed similar inhibition compared to free AMB.

### Antibiofilm Activity on the Mature Biofilm

**FIGURE 5:**
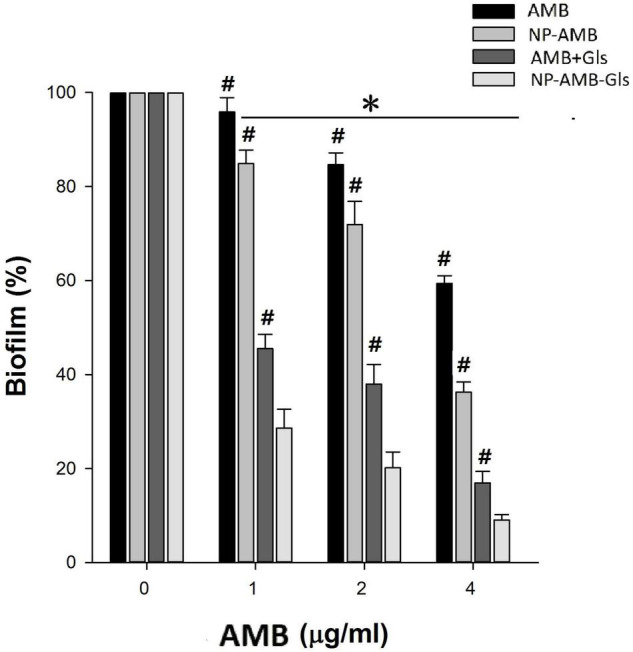
Eradication efficacy of NPs on the mature biofilm by *C. albicans* DAY 185 in a 96-well microplate. Mature biofilms were treated with different concentrations (0, 1, 2, and 4 μg/ml) of free AMB, AMB+Gls, CSNPs-AMB, and CSNPs-AMB-Gls for 24 h. The results represent the means and SDs (error bars), *n* = 3. Statistical significance was determined by the *t*-test analysis. *p < 0.05 for a comparison between the untreated and treated groups. #*p* < 0.05 for a comparison between CSNPs-AMB-Gls and other treated groups.

Due to the high resistance of the biofilm to antimicrobial agents, we used 4 × MIC for evaluating the antibiofilm activity of free AMB, AMB+Gls, CSNPs-AMB, and CSNPs-AMB-Gls on the mature biofilm (Figure 5). As expected, *C. albicans* DAY 185 in the biofilm increases resistance to free AMB or CSNPs-AMB without Gls compared to planktonic forms. However, both free AMB with Gls (AMB+Gls) and CSNPs-AMB-Gls showed a good extent of biofilm eradication. Moreover, CSNPs-AMB-Gls exhibited the highest activity in the eradication of preformed biofilm at any of the concentrations tested. At a concentration of 4 μg/ml AMB, AMB+Gls caused a 83.1% biofilm reduction, but CSNPs-AMB-Gls reduced the biofilm by 90.9%.

### Antibiofilm Efficacy on Silicone

**FIGURE 6:**
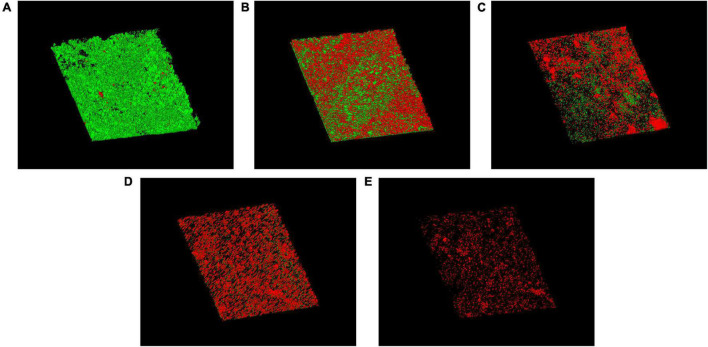
CLSM images of biofilms on untreated **(A)** or free AMB **(B)**, CSNPs-AMB **(C)**, AMB+Gls **(D)**, and CSNPs-AMB-Gls **(E)** treated silicone surfaces. Biofilms were stained with the Live/Dead^®^ BacLight™ Bacterial Viability and Counting kit. CLSM reconstructions show the three-dimensional staining pattern for live cells (SYTO-9, green) and dead cells (propidium iodide, red). Magnification, ×10.

Biofilm architecture and LIVE/DEAD organisms within the biofilm on silicone surfaces were investigated. The cell viability of the biofilm on silicone platelets treated with or without drugs was observed with CLSM (Figure 6). A large number of green-colored cells (live cells) were observed without any treatment (Figure 6A), which indicated that the mature biofilm was mainly composed of active cells. Treated with free AMB and CSNPs-AMB, the green-colored cells (live cells) were drastically reduced (Figures 6B,C). With the combination of Gls, more red-colored cells (dead cells) and less biofilm thickness were shown compared to free AMB treatment (Figures 6D,E).

**FIGURE 7:**
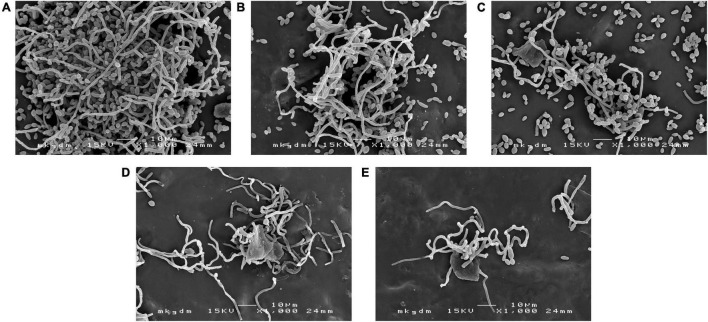
SEM images of the biofilm by *C. albicans* DAY 185 on untreated **(A)** or free AMB **(B)**, CSNPs-AMB **(C)**, AMB+Gls **(D)**, and CSNPs-AMB-Gls **(E)** treated silicone surfaces.

Scanning electron microscopy images confirmed the results mentioned above and revealed changes in the biofilm structures (Figure 7). In the control group, we can see the typical dense composition of biofilms (Figure 7A). However, treatment with free AMB and CSNPs-AMB significantly reduced colonization (Figures 7B,C). Furthermore, AMB+Gls and CSNPs-AMB-Gls resulted in only single cells or even free of cells on silicone platelets (Figures 7D,E), which meant that the biofilm structure was disrupted with the killing of *Candida* cells.

### Antibiofilm Activity Against Clinical Isolates

As shown in Supplementary Figure 1, biofilm formations by all clinical isolates were reduced in the presence of free AMB, AMB+Gls, CSNPs-AMB, and CSNPs-AMB-Gls (4 × MIC). CSNPs-AMB-Gls exhibited the best antibiofilm activity, which reduced 74.5, 85.2, and 59.5% biofilms of the clinical isolates BF1, BF2, and BF3, respectively.

## Discussion

Recently, NPs have been employed to protect antibiofilm agents from drug sequestration and improve penetration into the matrix, so as to enhance the therapeutic effect for treating biofilms. Here, CSNPs were functionalized with Gls and loaded with AMB to develop a new antibiofilm strategy until now.

In this work, AMB from CSNPs-AMB-Gls showed an initial burst release phase followed by a sustained release phase. This release mode is more suitable for the removal of biofilms. Because the initial burst release means a higher initial drug dose, which will reduce microbial drug tolerance (Cheow et al., 2010; Forier et al., 2014).

It has been reported that positively charged NPs could bind to negatively charged bacterial biofilm matrix components (Nafee et al., 2014; Baelo et al., 2015). Moreover, CS has the ability of penetrating into biofilms (Lu et al., 2014; Pu et al., 2014). Here, the proven ability to penetrate biofilms facilitates CSNPs to be a good drug carrier, which can carry drugs to penetrate into the biofilm as well and kill the inner cells.

For biofilm inhibition efficacy, Gls alone exhibited no obvious inhibition and CSNPs-AMB-Gls was not able to enhance the inhibition efficacy of biofilm formation relative to CSNPs-AMB. In addition, AMB alone or whenever driven with CSNPs (at all concentrations) showed similar efficacy on biofilm formation and planktonic cells, because the drug could kill planktonic fungi directly before biofilm formation.

In the preformed biofilm, our results exhibited a better eradication activity of CSNPs-AMB and CSNPs-AMB-Gls than free AMB and AMB+Gls, which indicated the superiority of NPs for biofilm treatment. NPs can protect the loaded antimicrobial agents from drug sequestration by the biofilm matrix (Forier et al., 2013; Koo et al., 2017). Moreover, NPs can deliver the drug into the biofilm matrix and directly target the microbial cells in the biofilm so as to maximize the therapeutic benefit. AMB+Gls and CSNPs-AMB-Gls showed a better detachment activity than free AMB and CSNPs-AMB, which suggested the synergistic efficacy of Gls.

β-1,3-glucan in *C. albicans* biofilm is considered to contribute to the resistance of antifungal drugs. As one of the important matrix components, β-1,3-glucan can protect cells in the biofilm by sequestering drugs (Nett et al., 2007). Similar functions of β-1,3-glucan have also been found in other *Candida* species (Tan et al., 2017). Therefore, the degradation of β-1,3-glucan resulted in the disruption of the biofilm matrix, and then improved AMB efficiency. This result is also consistent with our previous work (Tan et al., 2017, 2018). It can be ascribed to that Gls could disassemble the biofilm structure, which facilitates the mobility of positively charged NPs and binds to negatively charged biofilm components, thus providing high local concentrations of AMB to enhance the antibiofilm activity. Similarly, it has also been reported that the antibiofilm effect of antibiotic-containing NPs can be enhanced if the biofilm matrix was degraded by enzymes, such as DNase (Pu et al., 2014; Baelo et al., 2015). This interpretation could be further strengthened by SEM and CLSM images. CSNPs-AMB-Gls was more effective in killing *C. albicans* DAY 185 cells and disassembling the biofilm structure, which indicated the CSNPs-AMB-Gls better disrupted the biofilm matrix and thus increased killing of fungal cells.

Standardized and idealized laboratory conditions might make the microbe to lose some important pathophysiological characteristics when they are subcultured for decades (Fux et al., 2005). Thus, in our work, clinically isolated specimens were used to mirror clinical efficacy. The results demonstrate that CSNPs-AMB-Gls can disrupt clinically isolated biofilms as well as standard strain biofilms.

## Conclusion

In this study, we developed a biodegradable functional CSNP by loading AMB and Gls. CSNPs-AMB-Gls was homogeneously dispersed with a positive surface zeta potential. CSNPs-AMB-Gls was active in killing *C. albicans* cells and inhibiting biofilm formation, in addition to retaining the ability of Gls to disrupt the biofilm matrix. Excitingly, CSNPs-AMB-Gls exhibited the highest antibiofilm activity compared to free AMB and CSNPs-AMB in the mature biofilm. Although the assessment of biocompatibility and efficacy *in vivo* still needs to be evaluated, our studies pave the way for the application of CSNPs-AMB-Gls to treat *C. albicans* biofilm-related infections. Moreover, CSNPs-AMB-Gls can be employed as a platform to design more functions such as new drug delivery systems.

## Data Availability Statement

The original contributions and additional information presented in the study are included in the article/Supplementary Material, further inquiries can be directed to the corresponding author.

## Author Contributions

YT contributed to the conception of the study and finalized the manuscript. SM performed the experiment data and wrote the manuscript. JX contributed to analysis and manuscript preparation. RL helped to perform the analysis with constructive discussion. TD contributed to data analysis and manuscript editing. JL helped to edit the manuscript and evaluate the data. All authors contributed to the article and approved the submitted version.

## Conflict of Interest

The authors declare that the research was conducted in the absence of any commercial or financial relationships that could be construed as a potential conflict of interest.

## Publisher’s Note

All claims expressed in this article are solely those of the authors and do not necessarily represent those of their affiliated organizations, or those of the publisher, the editors and the reviewers. Any product that may be evaluated in this article, or claim that may be made by its manufacturer, is not guaranteed or endorsed by the publisher.

## Supplementary Material

Supplementary Figure 1 Antibiofilm activity against clinical isolates. The results represent the means and standard deviations (SDs; error bars) of three independent experiments. Statistical significance was determined by a *t*-test analysis. **p* < 0.05 for a comparison between the untreated and treated groups. #*p* < 0.05 for a comparison between chitosan nanoparticle (CSNP) loaded with Amphotericin B (AMB), which were functionalized with β-1,3-glucanase (Gls) (CSNP-AMB-Gls) and other treated groups.
