# Supplementary figures and images for: Enhancing the Antibiofilm Activity of β-1,3-Glucanase-Functionalized Nanoparticles Loaded With Amphotericin B Against Candida albicans Biofilm
Source: Front Microbiol. 2022 May 24;13:815091. doi: 10.3389/fmicb.2022.815091 (PMC9172620; doi:10.3389/fmicb.2022.815091)

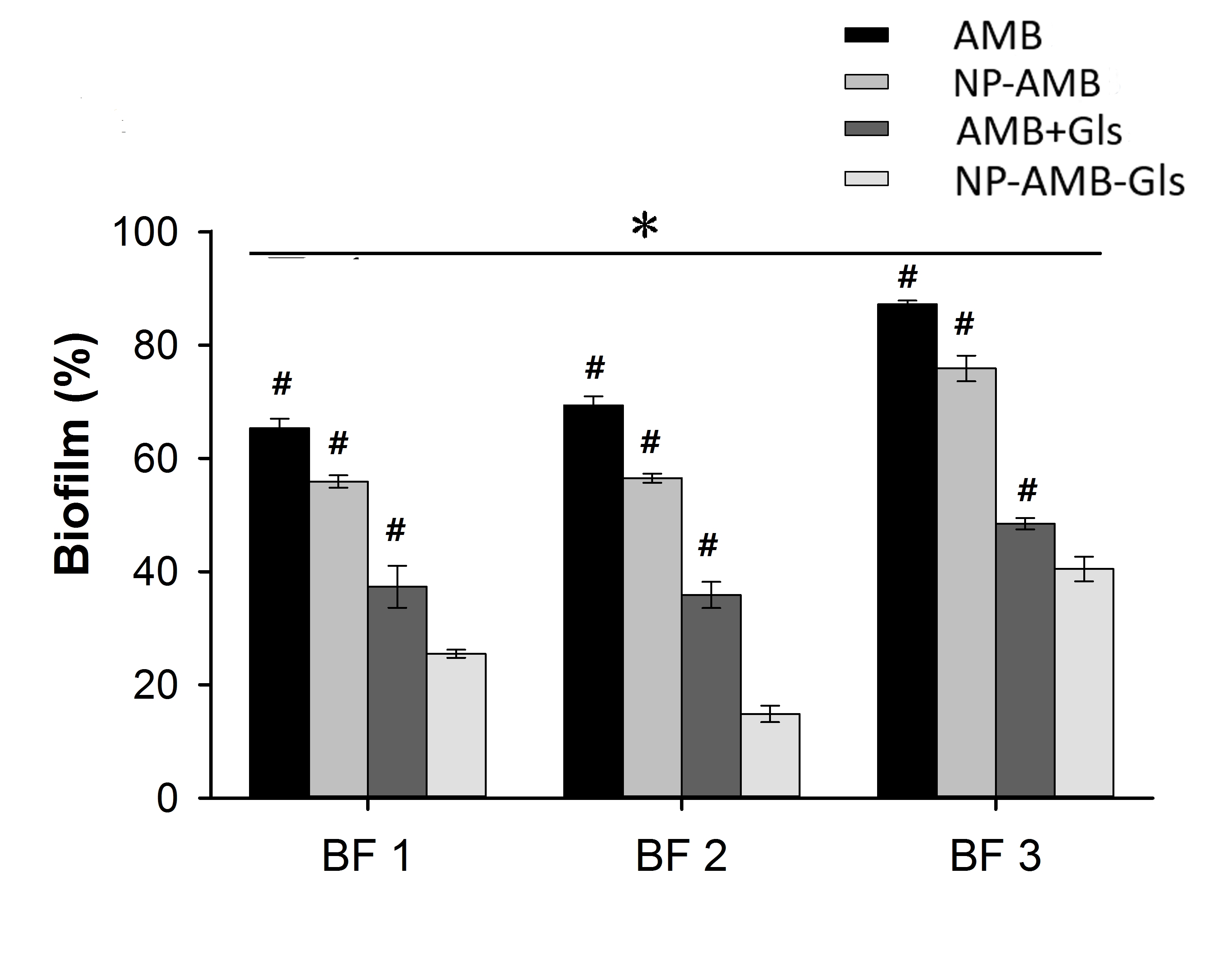

Supplement: Supplementary Figure 1 — Antibiofilm activity against clinical isolates. The results represent the means and standard deviations (SDs; error bars) of three independent experiments. Statistical significance was determined by a t-test analysis. *p < 0.05 for a comparison between the untreated and treated groups. #p < 0.05 for a comparison between chitosan nanoparticle (CSNP) loaded with Amphotericin B (AMB), which were functionalized with β-1,3-glucanase (Gls) (CSNP-AMB-Gls) and other treated groups. [file Image_1.JPEG]
